# Inhibition of the Sodium Calcium Exchanger Suppresses Alcohol Withdrawal-Induced Seizure Susceptibility

**DOI:** 10.3390/brainsci11020279

**Published:** 2021-02-23

**Authors:** Jamila Newton, Luli Rebecca Akinfiresoye, Prosper N’Gouemo

**Affiliations:** Department of Physiology and Biophysics, Howard University College of Medicine, Washington, DC 20059, USA; newton.jamila@gmail.com (J.N.); lakinfiresoye@gmail.com (L.R.A.)

**Keywords:** calcium signaling, generalized tonic–clonic seizures, KB-R7943, SN-6

## Abstract

Calcium influx plays important roles in the pathophysiology of seizures, including acoustically evoked alcohol withdrawal-induced seizures (AWSs). One Ca^2+^ influx route of interest is the Na^+^/Ca^2+^ exchanger (NCX) that, when operating in its reverse mode (NCX_rev_) activity, can facilitate Ca^2+^ entry into neurons, possibly increasing neuronal excitability that leads to enhanced seizure susceptibility. Here, we probed the involvement of NCX_rev_ activity on AWS susceptibility by quantifying the effects of SN-6 and KB-R7943, potent blockers of isoform type 1 (NCX1_rev_) and 3 (NCX3_rev_), respectively. Male, adult Sprague–Dawley rats were used. Acoustically evoked AWSs consisted of wild running seizures (WRSs) that evolved into generalized tonic–clonic seizures (GTCSs). Quantification shows that acute SN-6 treatment at a relatively low dose suppressed the occurrence of the GTCSs (but not WRSs) component of AWSs and markedly reduced the seizure severity. However, administration of KB-R7943 at a relatively high dose only reduced the incidence of GTCSs. These findings demonstrate that inhibition of NCX1_rev_ activity is a putative mechanism for the suppression of alcohol withdrawal-induced GTCSs.

## 1. Introduction

**Figure 1:**
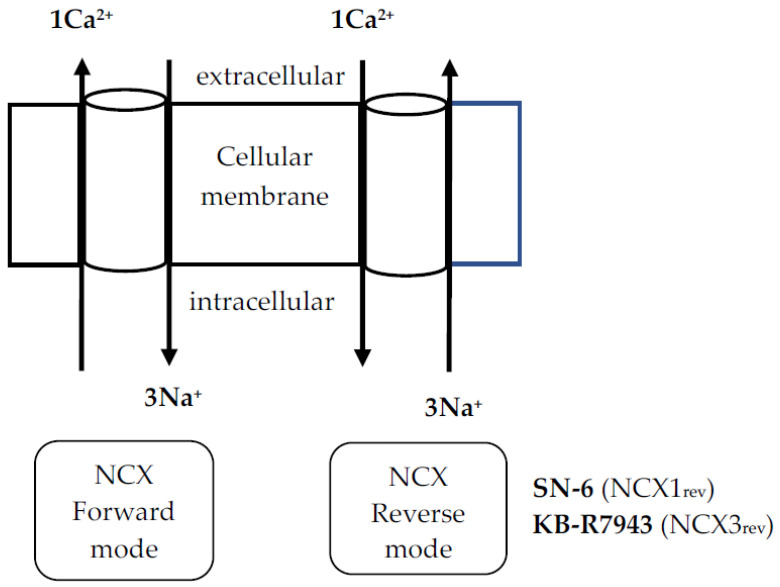
Schematic of Na^+^/Ca^2+^ exchanger (NCX) function in cells. 2-[[4-[(4-nitrophenyl)methoxy]phenyl]methyl]-4-thiazoli dinecarboxylic acid ethyl ester) and KB-R7943 (2-[2-[4-(4-nitrobenzyloxy)phenyl]ethyl] isothioureamethanesulfonate) are potent inhibitors of reverse mode activity for NCX1 and NCX3 isoform, respectively.

Seizures are the most common neurological deficits of the alcohol use disorder [1,2,3]; when associated with abrupt alcohol cessation, these seizures are referred to as “alcohol withdrawal-induced seizures” (AWSs). AWSs are usually generalized tonic–clonic seizures (GTCSs) that originate in the brainstem and can be resistant to modern anticonvulsants [4,5,6,7]. Therefore, there is a need to develop novel antiseizure therapies based on new putative mechanisms that can suppress alcohol withdrawal-induced neuronal hyperexcitability. Multiple lines of evidence indicate that increased intracellular Ca^2+^ levels ([Ca^2+^]_i_) contribute to the initiation, maintenance, and propagation of seizure activity [8,9,10]; in contrast, increasing extracellular Ca^2+^ levels can terminate epileptic activity [11,12]. Thus, inhibiting Ca^2+^ influx is a promising therapeutic approach for preventing seizures, including AWSs. In support of this hypothesis, blockade of the L-type of voltage-gated Ca^2+^ (Ca_V_) channels suppressed AWSs, including acoustically evoked AWSs [13,14,15]. In addition, AWSs have been associated with increases in synaptic activity of Ca_V_ currents, upregulation of L- and P-type Ca_V_ currents, and upregulation of L- and P/Q-type Ca_V_ channels [14,16,17,18,19,20]. However, the altered expression of L- and P-type Ca_V_ channels does not play a critical role in the initiation of acoustically evoked AWSs [14,17,19], suggesting that other Ca^2+^ entry routes may play a key role in the generation of AWSs. One Ca^2^+ entry route of interest is the Na^+^/Ca^2+^ (NCX), a bidirectional Ca^2+^ transporter that controls the level of intracellular Ca^2+^ [21,22]. There are three NCX isoforms, namely type 1 (NCX1), type 2 (NCX2), and type 3 (NCX3); each isoform has a distinct molecular expression pattern and pharmacological sensitivity [23,24,25,26,27,28]. For instance, NCX1 is distributed ubiquitously, NCX2 is mainly expressed in the brain and spinal cord, and NCX3 is found in the brain and skeletal muscle [23,24,25,26,27,28]. Nevertheless, these three NCX isoforms display several functional similarities [21,22,29]. In physiological conditions, NCX1-3 transports three Na^+^ into the cell in exchange for one Ca^2+^ (forward or direct mode activity) [21,22] Figure 1. However, NCX1-3 can also function in the reverse mode (NCX_rev_) by promoting Ca^2+^ influx and Na^+^ efflux [21,22] Figure 1. Such NCX_rev_-mediated Ca^2+^ influx may play important roles in the pathogenesis and pathophysiology of seizures. Accordingly, studies have shown that deletion of NCX1 suppressed the tonic flexion component of pentylenetetrazol (PTZ)-induced generalized clonic–tonic seizures [30]. In addition, pharmacological inhibition of NCX_rev_ activity reduced the incidence of seizures and the seizure severity in the model of acute seizures and inherited epilepsy [30,31,32,33]. Interestingly, upregulation and downregulation of NCX3 at both mRNA and protein levels were found in the hippocampus and dentate gyrus of mice subjected to chronic ethanol exposure for 30 days and 60 days, respectively [34]; whether these changes in NCX3 were associated with enhanced susceptibility to alcohol withdrawal-related GTCSs remains unknown. The role of NCX1 and NCX3 in the pathogenesis and pathophysiology of AWSs is not fully understood. Therefore, understanding the involvement of NCX1 and NCX3 in neuronal hyperexcitability and enhanced seizure susceptibility has important implications for preventing seizures, including AWSs. There are pharmacological tools that can be used to probe the role of NCX in the pathophysiology of seizures. Evidence indicates that SN-6 (2-[[4-[(4-nitrophenyl)methoxy]phenyl]methyl]-4-thiazoli dinecarboxylic acid ethyl ester) and KB-R7943 (2-[2-[4-(4-nitrobenzyloxy)phenyl]ethyl] isothioureamethanesulfonate) are inhibitors of the reverse mode activity of NCX but with potent and preferential activity on NCX1_rev_ and NCX3_rev_, respectively [25,35]. In this study, we probed the putative role of alcohol withdrawal-induced Ca^2+^ influx via NCX1_rev_ and NCX3_rev_ activities in the pathophysiology of AWSs by evaluating the efficacy of SN-6 and KB-R7943 to suppress acoustically evoked GTCS susceptibility in rats.

## 2. Materials and Methods

We used our rat model of alcohol withdrawal-induced seizure susceptibility because GTCSs in this model mimics the human condition [36,37,38].

### 2.1. Animals

Eight-week-old male Sprague–Dawley rats (*n* = 72; 250–320 g; Taconic, Germantown, New York, Unites States) were used for these experiments. Rats were housed in standard polycarbonate cages with standard chow and water ad libitum and maintained in a temperature- and humidity-controlled room on a 12/12-h light/dark cycle. All efforts were made to minimize the number of rats used in these experiments. All experimental procedures were approved by the Institutional Animal Care and Use Committee (Protocol MED-20-03) and were performed in accordance with the National Institutes of Health Guide for the Care and Use of Laboratory Animals [39].

### 2.2. Ethanol Administration

Ethanol intoxication and withdrawal were performed as previously described [14,17,18]. Briefly, ethanol solution (30%, *v*/*v*, from a 95% stock solution, U.S.P., The Warner-Gram Company, Cockeysville, Maryland, Unites States) in Isomil (Abbotts laboratory, Chicago, Illinois, United States) was administered by gavage injection three times per day (at 8-h intervals) for four days. The first dose of ethanol was 5 g/kg body weight, and subsequent doses were reduced and adjusted for each rat to achieve a moderate degree of intoxication that was determined based on a well-described intoxication scale [37,38]. Ethanol was withdrawn after the second dose on the fourth day. The average weight loss was <15%, and the mortality rate was ~14% (10 out of 72 rats) in the present study. In our model, blood ethanol concentrations were negligible at 24-h following the last dose of ethanol, when the seizure susceptibility peaked [14]. Thus, rats were tested for seizure susceptibility starting 22-h following ethanol withdrawal as previously done [14].

### 2.3. Acoustically Evoked Seizures and Pharmacological Treatment

To test for the role of NCX_rev_ activity on AWS susceptibility, we used SN-6 (2-[[4-[(4-nitrophenyl)methoxy]phenyl]methyl]-4-thiazoli dinecarboxylic acid ethyl ester, Tocris Bioscience, Ellisville, Missouri, United States) and KB-R7943 2-[2-[4-(4-nitrobenzyloxy)phenyl]ethyl] isothioureamethanesulfonate, Tocris Bioscience) potent inhibitor of NCX1_rev_ and NCX3_rev_, activity, respectively. Rats subjected to ethanol withdrawal were placed in an acoustic chamber, and an acoustic stimulus that consisted of pure tones (100–105 decibels sound pressure level; Med Associates, St Albans, Vermont, United States) was first presented until either seizure was elicited or 60 s passed with no seizure activity. Rats that did not respond to tones were tested again 1-h later using mixed sound at 110–120 decibels produced by an electrical bell. Acoustically evoked seizures following ethanol withdrawal consisted of wild running seizures (WRSs) that evolved into generalized tonic–clonic seizures (GTCSs). The seizure severity was classified into stages as follows: stage 0, no seizure in response to acoustic stimulus; stage 1, one episode of WRSs; stage 2, two or more episodes of WRSs; stage 3, one episode of WRSs followed by GTCSs; stage 4, two or more episodes of WRSs followed by GTCSs [14,37,38]. Rats subjected to ethanol withdrawal and exhibiting acoustically evoked seizures were subsequently used for pharmacological studies and randomly assigned into three groups: the vehicle-treated group (*n* = 8), SN-6-treated group (*n* = 7–8 per dose), and KB-R7943-treated group (*n* = 7–8 per dose). Both SN-6 and KB-R7943 were dissolved in sterile water containing 0.2% dimethyl sulfoxide using sonication (80 kHz, 100% power), filtered, and administered by oral gavage in a volume of 0.2 ml/100 g body weight using an 18-gauge stainless steel feeding needle. SN-6 and KB-R7943 were administered at the dose of either 1, 3, or 10 mg/kg based on previous in vivo pharmacological studies [30,31,32,33]. Rats were tested for AWSs 0.5, 1-, 2-, and 4-h following administration of the vehicle, SN-6 or KB-R7943; rats that did not display seizures were considered protected. Time intervals from the start of the acoustic stimulus and the onset of WRSs were recorded and referred to as seizure latency. For each animal, the occurrence of WRSs and GTCSs, and the seizure severity score were recorded.

### 2.4. Data Analysis

The investigators were blinded to group allocation during experiments and data analysis. The Origin 2021 software (Origin Northampton, Massachusetts, United States) was used for statistical analyses and to create graphs. The incidences of WRSs and GTCSs were analyzed using the Fisher’s Exact test, whereas the Kruskal–Wallis test (with Dunn’s post hoc correction) was used to analyze the seizure severity. To evaluate differences in seizure latency, two-way ANOVA followed by a Bonferroni post hoc correction was performed. The summary data are presented as median seizure score ± SEM, or percentage (%) for the incidence of WRSs and GTCSs. For all experiments, differences were considered significant at *p* < 0.05.

## 3. Results

### 3.1. Tremors 

The behavioral signs of ethanol withdrawal-induced hyperexcitability included tremors. All control-treated rats exhibited tremors up to 4-h after injections. Administration of SN-6 at the dose of 10 mg/kg significantly reduced the occurrence of tremors to 25% (*p* = 0.03, Fischer’s Exact test). SN-6 at the dose of 3 mg/kg did not notably reduced the incidence of tremors to 43% (*p* = 0.08). At the dose of 1 mg/kg, SN-6 did not considerably reduced the incidence of tremors to (71%, *p* = 0.47). KB-R7943 treatment also did not considerably reduced the incidence of tremors (1 mg/kg: 86%, *p* = 0.47; 3 mg/kg: 71%, *p* = 0.47; 10 mg/kg: 63%, *p* = 0.20). 

### 3.2. Acoustically Evoked Seizures

Twenty-two hours following ethanol withdrawal, rats were first tested for the susceptibility to acoustically evoked seizures consisting of WRSs that can progress into GTCSs. The incidence of AWS seizure susceptibility was 84% (52 out of 62 tested rats). Rats that exhibited AWSs were then randomly assigned to the control-treated group, SN-6-treated group, and KB-R7943-treated group. All control-treated rats (*n* = 8) had WRSs at all tested time-points (Figure 1). The incidence of GTCSs was 87.5%, 87.5, 75%, 87.5%, and 62.5% when tested at 0, 0.5-, 1-, 2-, and 4-h posttreatment, respectively (Figure 1).

### 3.3. Effects of SN-6 and KB-R7943 at the Dose of 1 mg/kg on the Expression of Acoustically Evoked Seizures

**Figure 2:**
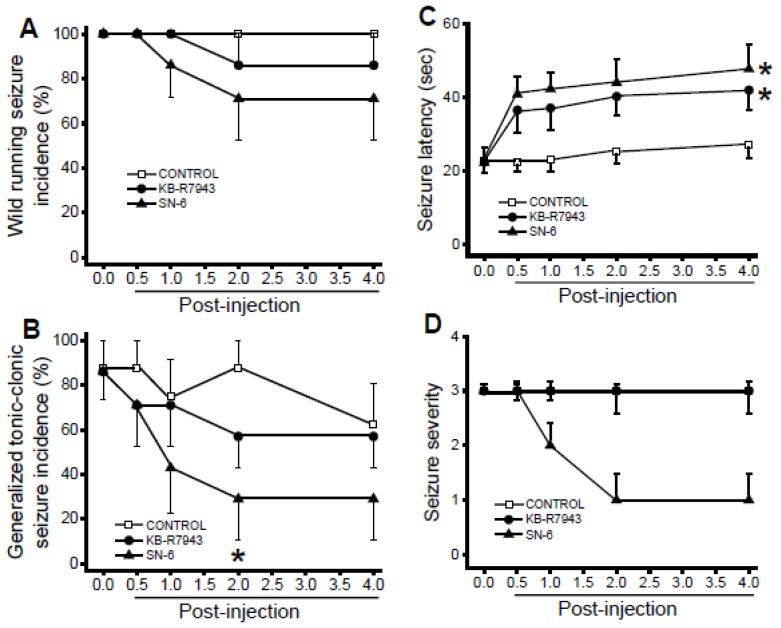
Effects of acute SN-6 or KB-R7943 treatment at the dose of 1 mg/kg on the occurrence of acoustically evoked alcohol withdrawal-induced seizure (AWS) susceptibility. The putative seizure suppressive effects of an inhibitor of NCX1_rev_ (SN-6, 1 mg/kg, p.o.) and NCX3_rev_ (KB-R7943, 1 mg/kg, p.o.) were evaluated at different posttreatment time points of 0.5-, 1-, 2-, and 4-h in rats exhibiting AWSs. (**A**). Both SN-6 and KB-R7943 treatments did not considerably reduce the incidence of WRSs component of AWSs. (**B**). SN-6 (but not KB-R7943) significantly reduced the incidence of GTCSs component of AWSs by the 2nd h post injection. (**C**). Both SN-6 and KB-R7943 treatments did not considerably delay the onset of seizure. (**D**). SN-6 did not considerably reduce the AWSs severity, while KB-7943 had no effect. Data from the incidence of WRSs and GTCSs are represented as mean percentage (%) ±SEM, Fisher’s Exact test was used for analysis. The seizure latency data are presented as mean ±SEM, and two-way ANOVA followed by Bonferroni post hoc correction was used for analysis. The seizure severity data were represented as median score ±SEM, and the Kruskal-Wallis test followed by Dunn’s post hoc correction was used for analysis. The summary of data was obtained from eight rats in the control-treated group and seven rats per group when SN-6 or KB-R7943 was used. * *p* < 0.05.

We first evaluated the effects of acute SN-6 and KB-R7943 treatment at a dose of 1 mg/kg (p.o.) on the occurrence of WRSs and GTCSs components of AWSs. Quantification showed that SN-6 and KB-R7943 did not considerably reduce the incidence of WRSs at all tested post injection time-points (Figure 2A). However, SN-6 (but not KB-7943) significantly reduced the incidence of GTCSs at the 2-h post injection time-point compared to the control-treated group (*p* = 0.04 Fisher’s Exact test, Figure 2B). We also evaluated the effects of SN-6 and KB-R7943 treatment on the latency to develop AWSs. In control-treated rats, the seizure latency was 22.75 ± 3.14 s (*n* = 8), 22.5 ± 2.58 s (*n* = 8), 23 ± 2.93 s (*n* = 8), 25.37 ± 3.39 s (*n* = 8), and 27.25 ± 3.74 s (*n* = 8), at 0, 0.5-, 1-, 2-, and 4-h post injection time-point, respectively (Figure 2C). For seizure latency, ANOVA revealed that the group means of time (F_(4.95)_ = 5.71, *p* = 0.0004) and treatment (F_(2,95)_ = 15.61, *p* = 0.00001) were significantly different. However, the interaction between time and treatment (SN-6 and KB-R7943) was not considerably different. Accordingly, SN-6 or KB-R7943 pretreatment slightly delayed the onset of AWSs at various tested post injection time-points compared to the control-treated group (Figure 2C). We also evaluated the extent to which SN-6 or KB-R7943 treatment at a dose of 1 mg/kg affected the severity of AWS. The median score of AWS was three across the tested time-points. We found that SN-6 did not considerably reduce AWS severity, while KB-R7943 had no effect when compared to the control-treated group (Figure 2D). Thus, SN-6 or KB-R7943 pretreatment at the dose of 1 mg/kg did not prevent the occurrence of acoustically evoked AWSs.

Next, we evaluated the effects of acute SN-6 or KB-R7943 pretreatment at a dose of 3 mg/kg on the expression of AWSs.

### 3.4. Effects of SN-6 and KB-R7943 at the Dose of 3 mg/kg on the Expression of Acoustically Evoked Seizures

**Figure 3:**
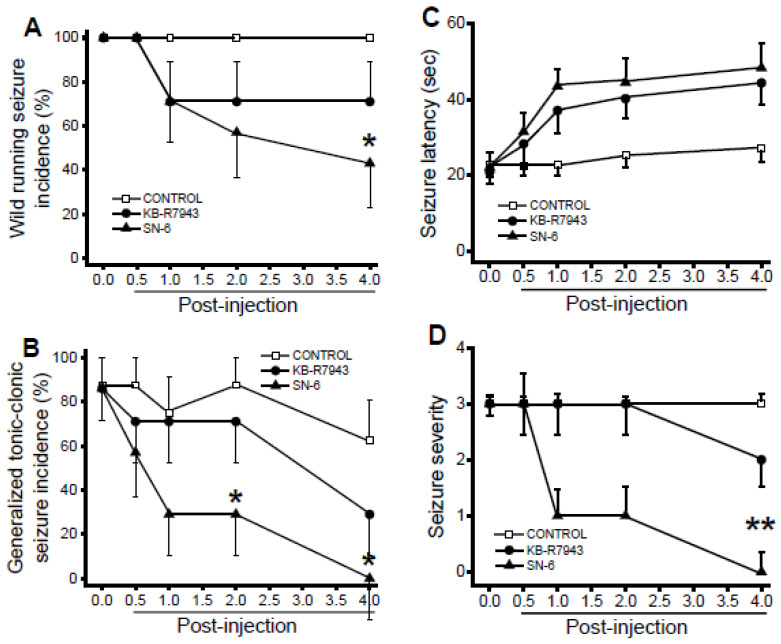
Effects of acute SN-6 or KB-R7943 treatment at the dose of 3 mg/kg on the occurrence of acoustically evoked AWS susceptibility. The putative antiseizure effects of the inhibitor NCX1_rev_ (SN-6, 3 mg/kg, p.o.) and NCX3_rev_ (KB-R7943, 3 mg/kg, p.o.) were evaluated at different posttreatment time points of 0.5-, 1-, 2-, and 4-h in rats exhibiting AWSs. (**A**). SN-6 (but not KB-R7943) treatment significantly reduced the incidence of WRSs component of WRSs. (**B**). SN-6 (but not KB-R7943) significantly reduced and suppressed the incidence of GTCSs component of AWSs by the 2nd and 4th h post injection, respectively. (**C**). Both SN-6 and KB-R7943 treatments not considerably delayed the onset of seizure. (**D**). SN-6 (but not KB-R7943) treatment suppressed the AWS severity. Data from the incidence of WRSs and GTCSs, seizure latency, and seizure severity are presented and analyzed as described in Figure 2. The summary of data was obtained from eight rats in the control-treated group and seven rats per group when SN-6 or KB-R7943 was used. * *p* < 0.05, ** *p* < 0.01.

In control-treated conditions, WRS occurred in all rats (*n* = 8) at all tested time-points (Figure 3A). The incidence of GTCSs was 87.5%, 87.5%, 75%, 87.5%, and 62.5% when tested at 0, 0.5-, 1-, 2-, and 4-h posttreatment, respectively (*n* = 8; Figure 3B). Quantification showed that SN-6 significantly reduced the incidence of WRSs at the 4-h posttreatment time-point, when compared to the control-treated group (*p* = 0.04 Fisher’s Exact test; Figure 3A). KB-R7943 treatment, however, did not considerably reduce the incidence of WRSs when compared with the control-treated group (Figure 3A). SN-6 pretreatment also significantly reduced the incidence of GTCSs at 2-h (*p* = 0.04 Fisher’s Exact test) and 4-h (*p* = 0.02 Fisher’s Exact test) posttreatment time-points compared to the control-treated group (Figure 3B). The suppression of GTCSs by SN-6 pretreatment was associated with a significant reduction in the severity of AWSs at the 4-h post injection time-point, compared to the control-treated group (z = 3.02, *p* = 0.01 Dunn’s correction; Figure 3D). KB-R7943 pretreatment only slightly reduced the severity of AWSs (Figure 3D). For the seizure latency analysis, ANOVA reveals that the mean group of time (F_(4.95)_ = 6.67, *p* = 0.00001) and treatment (F_(2,95)_ = 16.61, *p* = 0.00001) are significantly different. However, the interaction between time and treatment (SN-6 or KB-R7943) was not significant. Accordingly, we found that the anticonvulsant effect of SN-6 was not associated with considerable delay in the onset of seizure compared to the control-treated group (Figure 3C). Finally, we evaluated the extent to which acute SN-6 and KB-R7943 pretreatment at a dose of 10 mg/kg alters the expression of acoustically evoked AWSs.

### 3.5. Effects of SN-6 and KB-R7943 at the dose of 10 mg/kg on the Expression of Acoustically Evoked Seizures

**Figure 4:**
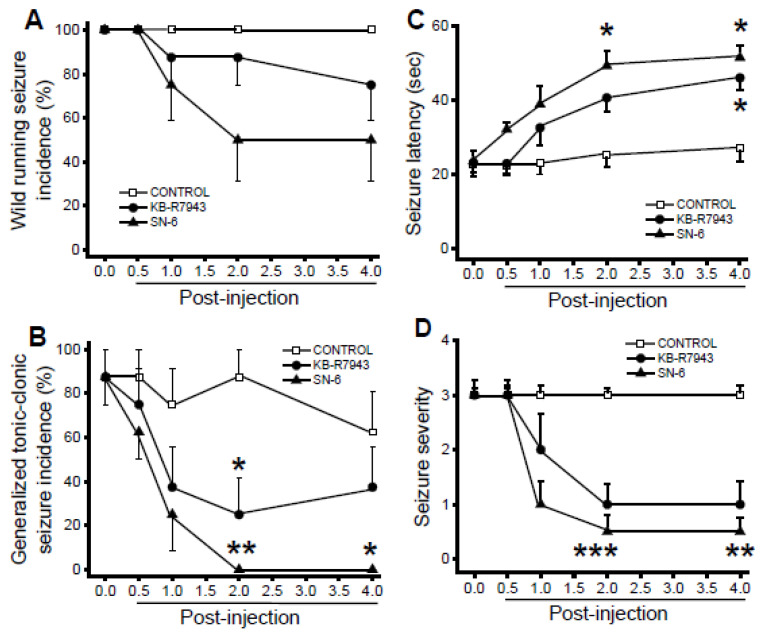
Effects of acute SN-6 or KB-R7943 treatment at the dose of 10 mg/kg on the occurrence of acoustically evoked AWS susceptibility. The putative seizure suppressive effects of the inhibitor NCX1_rev_ (SN-6, 10 mg/kg, p.o.) and NCX3_rev_ (KB-R7943, 10 mg/kg, p.o.) were evaluated in rats exhibiting AWSs. (**A**). Both SN-6 and KB-R7943 treatments did not considerably reduce the incidence of WRSs component of AWSs. (**B**). SN-6 and KB-R7943 treatment completely suppressed and reduced the occurrence of GTCSs component of AWSs, respectively, by the 2nd h post injection. (**C**). SN-6 and KB-7943 treatment delayed the onset of AWS by the 2nd and 4th h posttreatment, respectively. (**D**). SN-6 (but not KB-R7843) significantly reduced the AWSs severity. Data from the incidence of WRSs and GTCSs, seizure latency, and seizure severity are presented and analyzed as described in Figure 2. The summary of data was obtained from eight rats per group. * *p* < 0.05, ** *p* < 0.01, *** *p* < 0.001.

Administration of either SN-6 or KB-R7943 did not considerably reduce the incidence of the WRSs component of AWSs at all tested time-points (Figure 4A). However, SN-6 pretreatment completely suppressed the incidence of GTCSs 2-h (*p* = 0.01 Fisher’s Exact test) and 4-h (*p* = 0.04 Fisher’s Exact test) post injection time-points compared to the control-treated group (Figure 4B). Pretreatment with KB-R7943 also significantly reduced the incidence of GTCSs at the 2-h post injection time-point (*p* = 0.04 Fisher’s Exact test; Figure 3B). For the analysis of seizure latency, ANOVA revealed that group means of time (F_(4.105)_ = 15.87, *p* = 0.00001) and treatment (F_(2,105)_ = 24.10, *p* = 0.00001) were significantly different. In addition, the interaction between time and treatment (SN-6 or KB-R7943) was also significant (F_(8,105)_=2.57; *p* = 0.01). Accordingly, SN-6 pre-treatment significantly delayed the onset of AWSs at 2-h (*P* = 0.0005, Bonferroni correction) and 4-h (*p* = 0.0005, Bonferroni correction) post injection time-points, when compared to the control-treated group (Figure 4D). KB-R7943 pretreatment also significantly delayed the onset of seizures 2-h post injection time point (*p* = 0.02 Bonferroni correction, Figure 4C). The suppressive effect of SN-6 (but not KB-R7943) was associated with a significant reduction in the seizure severity at 2-h (z = 3.45, *p* = 0.001 Dunn’s correction), and 4-h (z = 3.34, *p* = 0.003 Dunn’s correction) post injection time-point (Figure 4D).

## 4. Discussion

In this study, we evaluated the inhibition of NCX1_rev_ and NCX3_rev_ activities as a potentially novel mechanism underlying the increased susceptibility to acoustically evoked AWSs. We found that inhibiting NCX1_rev_ activity completely suppressed the occurrence of GTCSs component of AWSs, reduced the AWS severity, and delayed the onset of AWSs. We also found that blocking NCX3_rev_ activity significantly reduced the incidence of GTCSs and delayed the occurrence of AWSs. Together, these findings suggest that: (i) inhibiting NCX1_rev_ activity is sufficient to suppress GTCS susceptibility following alcohol withdrawal and may play a role in the pathogenesis of these seizures; (ii) altered NCX3_rev_ activity only reduced the incidence of GTCS, suggesting that this NCX isoform may not play a major role in the pathogenesis of these seizures. The delayed onset of acoustically evoked AWSs, following inhibition of NCX1_rev_ and NCX3_rev_ activities, may reflect the inhibitory effect on the propagation of seizure activity from the inferior colliculus, the seizure initiation site, to brain sites implicated in the generation of GTCSs [36,40,41]. The main function of NCX_rev_ activity is to transport one Ca^2+^ into the neuron in exchange for three Na^+^ [21,22]. Therefore, the suppression of acoustically evoked AWSs following the inhibition of NCX_rev_ activity suggests that inhibiting Ca^2+^ entry via NCX_rev_ activity is a potential anticonvulsant mechanism. However, the mechanisms underlying the anticonvulsant effect of inhibiting NCX_rev_ activity are not completely elucidated. NCX_rev_ activity may be driven by Na^+^ influx, thus triggering massive Ca^2+^ entry while promoting the export of Na^+^ [42]. The resulting secondary [Ca^2+^]_i_ increase can activate various Ca^2+^-dependent mechanisms, including Ca^2+^-activated K^+^ channels and Ca^2+^-activated chloride channels. This hypothesis is partially unlikely, as activation of Ca^2+^-activated K^+^ channels and Ca^2+^-activated chloride channels hyperpolarize the membrane and, therefore, increase the seizure threshold. Alternatively, we postulated that altering the electrogenic effect of NCX_rev_ activity might contribute to the anticonvulsant effect of NCX inhibitors [32].

Multiple lines of evidence indicate that NCX may play important roles in the pathophysiology of seizures. Accordingly, genetic deletion of NCX1 suppressed the occurrence of pentylenetetrazole (PTZ)-induced tonic seizures in mice [30]. In the rat PTZ model of generalized seizures, administration of either SN-6 or KB-R7943 reduced the incidence of both clonic and clonic–tonic seizures component of PTZ-induced generalized seizures [32]. SN-6 and KB-R7943 also reduced the severity of PTZ-induced seizures; detailed analysis revealed that SN-6 suppressed motor seizures but not limbic seizures [32]. In line with these findings, administration of KB-R7943 also suppressed pilocarpine-induced recurrent clonic–tonic seizures but not limbic seizures [31]. Together, these findings suggested that inhibiting NCX_rev_ activity can suppress PTZ-induced clonic and tonic seizures, but not limbic seizures. In the genetically epilepsy-prone rat (GEPR-3), a model of inherited epilepsy characterized by the occurrence of acoustically evoked WRSs and GTCSs, SN-6 administration markedly reduced the incidence of WRSs and GTCSs, as well as the seizure severity [33]. In the present study, we found that SN-6 completely prevented the occurrence of alcohol withdrawal-induced GTCSs and suppressed AWS severity. Together, these findings suggested that alcohol withdrawal-induced GTCSs are more sensitive to the inhibition of NCX1_rev_ than inherited GTCSs in the GEPR-3s. Interestingly, both alcohol withdrawal-induced WRSs and inherited WRSs in the GEPR-3s were resistant to the inhibition of NCX_rev_ activity. It is tempting to speculate that inhibiting NCX_rev_ activity markedly suppresses GTCSs but modestly alters the occurrence of complex seizures, such as WRSs.

## 5. Conclusions

In summary, inhibition of NCX1_rev_ activity suppresses the occurrence of alcohol withdrawal-induced GTCSs and reveals a putative novel mechanism for the suppression of AWSs.

## Data Availability

Data are available upon request from the corresponding author.
